# *Staphylococcus aureus* protein A causes osteoblasts to hyper-mineralise in a 3D extra-cellular matrix environment

**DOI:** 10.1371/journal.pone.0198837

**Published:** 2018-06-21

**Authors:** Nicola Kavanagh, Fergal J. O’Brien, Steve W. Kerrigan

**Affiliations:** 1 Cardiovascular Infection Research Group, Irish Centre for Vascular Biology, Royal College of Surgeons in Ireland, Dublin, Ireland; 2 Tissue Engineering Research Group, Department of Anatomy, Royal College of Surgeons in Ireland, Dublin, Ireland; 3 Trinity Centre for Bioengineering, Trinity Biomedical Sciences Institute, Trinity College Dublin, Dublin, Ireland; 4 Advanced Materials and Bioengineering Research (AMBER) Centre, Royal College of Surgeons in Ireland and Trinity College Dublin, Dublin, Ireland; 5 School of Pharmacy and Molecular and Cellular Therapeutics, Royal College of Surgeons in Ireland, Dublin, Ireland; University of Notre Dame, UNITED STATES

## Abstract

Osteomyelitis is an inflammatory bone infection that is caused most commonly by the opportunistic pathogen *Staphylococcus aureus*. Research into staphylococcal induced bone infection is typically conducted using traditional 2D *in vitro* culture settings, which is not fully representative of the dynamic *in vivo* environment. In this study we utilised a collagen glycosaminoglycan scaffold, previously developed for bone tissue engineering, as a representative 3D model of infection. The scaffold resisted degradation and retained its pore structure, which is important for cellular function and survival, when seeded with both cells and bacteria. Using this model, we showed that in the presence of *S*. *aureus*, osteoblast proliferation was reduced over 21 days. Interestingly however these cells were more metabolically active compared to the uninfected cells and demonstrated increased mineralisation. Protein A (SpA) is a virulence factor found on the surface of *S*. *aureus* and has been shown to interact with osteoblasts. When SpA was removed from the surface of *S*. *aureus*, the osteoblasts show comparable activity with the uninfected cells—demonstrating the importance of SpA in the interaction between bone cells and *S*. *aureus*. Our results suggest that infected osteoblasts are capable of over-compensating for bone loss and bone destruction by increasing mineralisation in a 3D environment, key elements required for ensuring bone strength. It also reinforces our previously established result that *S*. *aureus* SpA is a critical mediator in osteomyelitis and might be a potential novel drug target to treat osteomyelitis by preventing the interaction between *S*. *aureus* and osteoblasts.

## Introduction

Osteomyelitis is characterised by the presence of an infection in the typically sterile bone marrow, in particular cortical bone and periosteum. It is characterised by severe non-resolving inflammation coupled with progressive bone loss and bone destruction [1, 2]. There are a number of opportunistic pathogens that are capable of causing osteomyelitis including bacteria, fungus and virus, however epidemiologic data suggest bacteria are the biggest offenders [3].

Opportunistic bacteria use a number of different mechanisms to gain entry to the otherwise sterile sites in the bone. For example, bones are highly vascularised where arterial blood travels through the endosteal cavity to the marrow sinusoids before exiting through small vessels in the cortex [4]. These bacteria in the blood (bacteraemia) can migrate through the vascular endothelial bed into the bone space thus initiating what is termed haematogenous osteomyelitis. Haematogenous osteomyelitis most commonly affects children and is primarily caused by *Staphylococcus aureus* and, the gram-negative enteric bacteria *Escherichia coli* [5]. Bacteria may also gain entry to the bone due to infection spread from a distant site. Contiguous-focus osteomyelitis without vascular insufficiency can form from contaminated wounds or infected sites of implanted prosthetic material. Contaminated wounds, usually due to trauma, are often polymicrobial however infected sites due to a prosthetic material are often caused by Staphylococci [6]. Finally, a contiguous-focus osteomyelitis with vascular insufficiency typically arises from foot ulcers due to diabetes, where a non- resolving wound becomes infected, and often polymicrobial in nature, however predominated by Staphylococci [7]. Regardless of the source of the contiguous-focus osteomyelitis, the opportunistic bacteria are in close proximity with bone to which they can attach, triggering bone loss and destruction.

*S*. *aureus* is responsible for up to 75% of osteomyelitis cases [8]. In healthy individuals *S*. *aureus* permanently colonizes the anterior nares of the nostrils of about 20% of the population and is transiently associated with the rest [9]. The success of *S*. *aureus* as a pathogen in osteomyelitis is attributed to its ability to infect the bone forming cells, osteoblasts and bone resorbing cells, osteoclasts. Recent work from our lab by Claro *et al*., demonstrated that the major *S*. *aureus* cell wall protein, protein A is responsible for binding to and inducing the signals that trigger bone loss and destruction [10–12]. These studies were carried out using a two dimensional (2D) cell culture based assay. The physiological relevance of traditional 2D cell culture systems have come under increased scrutiny as they do not physiologically represent the complex dynamic environment in which cells are found in the human body [13–15]. To address the growing concern for more representative models without using animals, there has been an emergence of 3D models using tissue engineered biomaterials across various disciplines including cardiovascular disease, cancer, neurology and infection [16–18]. A biomaterial commonly used in 3D systems is collagen, a biodegradable, biomimetic and biocompatible ECM protein. In our lab, we have been using collagen-based scaffolds initially developed for regenerative medicine applications as advanced 3D pathophysiology *in vitro* systems for drug development and screening and for studying cellular crosstalk in co-cultures and understanding disease states in cancer, angiogenesis, immunology and infection [19–21].

The aim of this study was to develop a 3D model of bone infection to better develop our understanding of the molecular and functional responses of bone cells to infection. Achieving this we developed a 3D model of bone infection, using a collagen glycosaminoglycan biomaterial, which better represents the physiological bone microenvironment, in which bone cells, specifically osteoblasts, are found. We demonstrate that *S*. *aureus* significantly reduced osteoblast proliferation. Most interestingly, the remaining osteoblasts were more metabolically active, and resulted in hyper mineralisation which we believe is a counter measure by the osteoblasts to compensate for the loss of bone and bone destruction. Our results demonstrate key events not shown previously in 2D which are critical for understanding the progression of bone infection using a 3D model. A better understanding of the molecular interactions and cellular responses using 3D models of bone infection is critical for the development of novel therapeutics to treat this debilitating disease.

## Materials and methods

### Bacterial strains and growth conditions

The *S*. *aureus* strains used in the functional study are Newman wildtype (WT), Newman ΔSpA and Newman pCu1 (SpA+). All strains were cultured in Brain Heart Infusion (BHI) broth (Sigma-Aldrich, Wicklow, Ireland) and incubated statically at 37°C for 18 h. *S*. *aureus* strain pCu1 (SpA+) was cultured in BHI containing 10 μg/ mL chloramphenicol (Sigma-Aldrich, Wicklow, Ireland). In some studies *S*. *aureus* were fixed in 4.8% formaldehyde (Sigma-Aldrich, Wicklow, Ireland) under constant agitation, centrifuged at 4, 000 *g* for 10 min and washed 3x times using phosphate buffered saline (PBS) (Sigma-Aldrich, Wicklow, Ireland). Bacteria were adjusted to 1 x 10^8^ or 1 x 10^9^ CFU/ mL using a spectrophotometer (Libra S21/S22, United Kingdom).

### Protein A expression on the surface of *Staphylococcus aureus*

A dot blot was used to determine expression of Protein A on the surface of *S*. *aureus*. Bacterial cultures were centrifuged at 4000 *g* for 10 min, resuspended in PBS and set to 1 x 10^8^ CFU/ mL. 10 μL of the bacterial suspension was dotted onto a nitrocellulose membrane and left to dry prior to blocking in 10% milk in PBS containing 1% trition (PBST). Monoclonal anti-protein A antibody (Sigma-Aldrich, Wicklow, Ireland) was added at a concentration of 1:1000 overnight at 4°C. The membrane was washed 3x times using PBST and the secondary antibody goat anti-mouse antibody was added at a concentration of 1:3000 for 1 h at room temperature. The membrane was finally washed 3x times using PBST and developed using ECL kit chemical (EMD Millipore, Massachusetts, USA).

### Cell culture conditions

The mouse clonal pre-osteoblastic cell line, MC3T3 –E1 (ATCC, Middlesex, UK) was used throughout these studies. Cells were cultured in T175 tissue culture flasks (Sarstedt, Wexford, Ireland) containing α-MEM supplemented with 10% foetal bovine serum (FBS) (Biosera Ltd., East Sussex, UK), 2% penicillin-streptomycin and 1% L-glutamine (Sigma-Aldrich, Wicklow, Ireland) and incubated at 37°C and 5% CO_2_. For live studies, penicillin-streptomycin was omitted from the media. When the osteoblasts were 70–80% confluent they were harvested using trypsin EDTA (Sigma-Aldrich, Wicklow, Ireland), re-suspended in growth medium and counted using a haemocytomer.

### Preparation of collagen glycosaminoglycan scaffolds

The collagen glycosaminoglycan (CG) suspension contained 1.8 *g* microfibrillar type I collagen from bovine tendon (Integra Life Sciences, New Jersey, USA) and 0.16 *g* chondroitin 6 sulphate blended at 15, 000 rpm for 90 min in 360 mL 0.05 M acetic acid (Sigma-Aldrich, Wicklow, Ireland) using an overhead blender at a constant temperature of 4°C (Ultra Turrax T18 OverheadBlended, IKAWorks Inc.,USA). Previously, we demonstrated that larger pore sizes were optimal for bone formation. Freeze-drying the CG suspension to a temperature of -10°C generated scaffolds with a large pore size of 325 μM [22]. The CG scaffolds were sterilised using dehydrothermal treatment (DHT) in a vacuum oven (VacuCell, MMM, Germany). DHT is also a biophysical crosslinking treatment which enhances the mechanical properties of collagen. To further improve the mechanical properties of the biomaterial, the chemical crosslinker 1-Ethyl-3-dimethyl aminopropyl carbodiimide (EDAC) and N-hydroxysuccinimide (NHS) (Sigma-Aldrich, Wicklow, Ireland) was used in addition to DHT [23].

### Seeding of the collagen glycosaminoglycan scaffold

Scaffolds from CG sheets were obtained using a biopsy punch and placed into a 24 well suspension plate (Sarstedt, Wexford, Ireland). The top surface of each of the scaffold was seeded with 10 μL of half the cell suspension (2.5 x 10^5^ / 1 x10^6^ cells). The 24 well plates were then placed in an incubator for 15 min to allow initial cellular attachment. The scaffolds were then turned over and the opposite surface was seeded with 10 μL of the remaining cell suspension (2.5 x 10^5^ / 1 x10^6^ cells) and incubated for a further 15 min. After the second incubation period, 2 mL α-MEM medium was added to each well and the plates were returned to the incubator.

### Infection of collagen glycosaminoglycan scaffolds

Media was removed from the wells and replaced with either live *S*. *aureus* or S. aureus fixed in a 4.8% formaldehyde solution. When adding *S*. *aureus* Newman to the seeded scaffolds, α-MEM media was aspirated from the CG scaffold and 1 mL fixed S. aureus Newman was added to the well. The infected scaffolds were incubated at 37°C and 5% CO_2_ for 1 h. To maintain osteoblast growth on the seeded scaffolds, pre-warmed α-MEM was added to the well post 1hr incubation. The α-MEM media was changed every 2–3 d until the end of the infection period.

### Determining collagen content via hydroxyproline analysis in the presence of live *Staphylococcus aureus* infection

Previously, we have demonstrated that the mechanical properties of the scaffolds significantly influence cell attachment, proliferation and migration [23]. As collagen is the main component of the scaffolds, collagen degradation in the presence of live bacteria was determined by the hydroxyproline assay using the protocol described in Kafienah and Sims [24]. Briefly, samples were mixed with 38% hydrochloric acid and incubated at 110°C for 18 h to allow hydrolysis to occur. Thereafter the samples were dried in a fume hood overnight and the sediments were suspended in ultra-purewater. Chloramine T and 4-(dimethylamino)benzaldehyde (Sigma-Aldrich, Wicklow, Ireland) were added and the hydroxyproline content quantified with a trans-4-hydroxy-l-proline (Fluka analytical, Switzerland) standard using a Synergy™ HT (BioTek Instruments Inc, Vermont, USA) multi-detection microplate reader at a wavelength of 570 nm. Hydroxyproline content was determined from the calibration curve obtained from the hydroxproline standard concentrations and the collagen content was calculated using a value of hydroxyproline to collagen ratio of 1:769. Each biochemical constituent was normalized to the tissue dry weight [25].

### Scanning Electron Microscopy (SEM) analysis of pore architecture

Scaffold pore architecture was visualized using Scanning Electron Microscopy (SEM). Scaffolds were harvested at days 1, 3, 5 and 7 days post infection were stored in 3% glutaraldehyde (Sigma-Aldrich, Wicklow, Ireland) at 4°C. Samples were processed using critical point drying (CPD). CPD is a process used to dehydrate biological samples without affecting the structure of the sample due to the lack of surface tension prior to SEM. After CPD, the samples were mounted in carbon cement for increased electrical conductivity and coated in Gold Palladium. The samples were imaged using Zeiss Ultra Plus SEM at 5KV with a SE2 detector (Zeiss, Oberkochen, Germany).

### Confocal microscopy to determine cellular and bacterial infiltration of the scaffolds

Collagen scaffolds were removed from culture 1 day post infection to ensure that osteoblasts were still viable. Scaffolds were processed, embedded in paraffin wax and sectioned into 10 micron slices using a microtome (Leica RM 2255; Leica, Vertrieb,Germany). The slices of scaffolds were mounted onto a glass slide and left to fix in an oven set to 60°C overnight. *S*. *aureus* was stained using FITC anti-staphylococcal antibody (Abcam, Cambridge, UK) at a concentration of 1:1000 overnight at 4°C in a humidified environment. Osteoblasts were stained with 4', 6-diamidino-2-phenylindole (DAPI) (Invitrogen, Massachusetts, USA) following mounting on the coverslip. Images were acquired using confocal microscopy (Zeiss 710 NLO, Germany) following excitation at 488 nm, and emission at > 500 nm.

### Quantification of osteoblast cell number in the presence and absence of *Staphylococcus aureus*

PicoGreen (Invitrogen, Massachusetts, USA) was used to measure DNA concentration as a measure of osteoblast proliferation. Osteoblasts were lysed on the scaffolds using 0.2 M lysis buffer containing triton X-100 followed by 3x freeze thaw cycles. Lysed samples were added to a 96 well plate along with PicoGreen reagent. All samples were measured using Varioskan Flash multimode plate reader immediately after the addition of the PicoGreen reagent (Fisher Scientific, Dublin, Ireland). Fluorescence for PicoGreen was measured at excitation 485 nm and emission 538 nm. DNA concentration was deduced using a standard curve.

### Determining osteoblast viability in the presence and absence of *Staphylococcus aureus*

Alamar Blue (Thermofisher, Dublin, Ireland) was used to measure cell viability. At days 0, 7, 14 and 21 post addition of *S*. *aureus*, Alamar Blue was added to the osteoblast media to a final concentration of 10% of the final volume. The plate was agitated using an orbital rotator for 4 h at 70 rpm, at 37°C in 5% CO_2_. After 4 h, 100 μl of the media was removed, added into black 96 well plates and fluorescence was read at a wavelength 560 nm for excitation and 590 nm for emission on a spectrophotometer (Varioskan Flash, Thermofisher, Ireland).

### Quantification of alkaline phosphatase activity in the presence of *Staphylococcus aureus*

Para-NitroPhenyl Phosphate (Anaspec, California, USA) was used to measure alkaline phosphatase. To measure intracellular alkaline phosphatase, scaffolds at days 0, 7, 14 and 21 post addition of *S*. *aureus* were removed and suspended in lysis buffer (0.1 M sodium acetate buffer and 2% triton X-100). To measure extracellular alkaline phosphatase, the culture media was aspirated from the well at days 0, 7, 14 and 21 after the addition of *S*. *aureus* and incubated with 10 mM para-nitrophenyl Phosphate for 30–60 min. Photometric absorbance was read at A_405nm_ on a spectrophotometer (Varioskan Flash, Thermofisher, Ireland).

### Determining osteoblast mineralisation by measuring calcium production

StanBio Calcium Assay (Invitrogen, Massachusetts, USA) was used to measure calcium deposition, or mineralisation in the scaffolds. Quantification was carried out according to the manufacturer’s instructions. Briefly, at days 0, 7, 14 and 21 post addition of *S*. *aureus*, scaffolds were incubated at 4°C in 0.5 M HCL overnight. Following this incubation, 10 μL of each sample was added to a 96 well plate and 200 μL of the chromogenic reagent working solution was added to the samples. Photometric absorbance was read at A_596nm_ on a spectrophotometer (Varioskan Flash, Thermofisher, Ireland).

### Statistical analysis

Statistical analysis was determined by using Graph Pad software. The representative N numbers represent a technical triplicate over several independent experiments. The statistical differences between two groups were calculated by un-paired Student’s t-test and two way analysis of variance (ANOVA) with Tukeys post hoc test. Data were represented as the mean values ± the standard error of the mean (SEM). Statistical significance was declared at *P*<0.05.

## Results

### Scaffolds crosslinked with DHT and EDAC resists collagen degradation when co-cultured with both osteoblasts and *Staphylococcus aureus*

**Fig 1:**
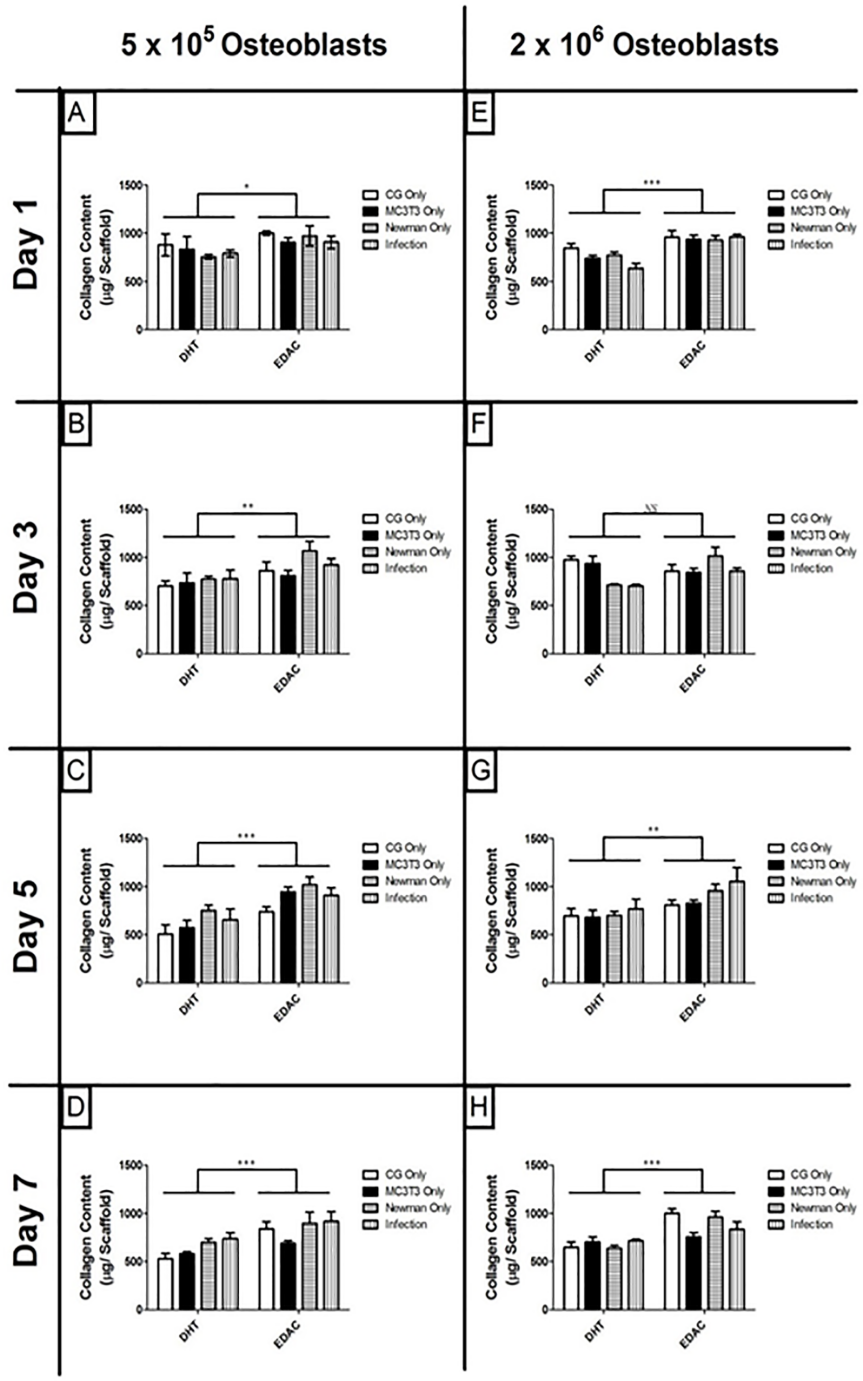
The collagen content of scaffolds following infection by *Staphylococcus aureus* for 7 days. Scaffolds were seeded with 5 x 10^5^ osteoblasts and infected with 1 x 10^8^ *S*. *aureus* (A-D) and 2 x 10^6^ osteoblasts infected with 1 x 10^9^ *S*. *aureus* (E-H), hydrochloric acid boiled and hydroxyproline content measured. Scaffolds across both seeding densities crosslinked using DHT only have significantly reduced collagen content compared to scaffolds crosslinked using DHT /EDAC * P < 0.05, ** P< 0.005, *** P < 0.0001 (N = 3).

Pore architecture is critical for cellular survival in 3D collagen scaffolds (30). *S*. *aureus* is able to produce many proteases capable of degrading collagen, which can significantly affect the stability and architecture of the scaffold [26]. Therefore we first sought to investigate if the presence of live *S*. *aureus* infection could degrade collagen in the scaffolds. Scaffolds are cross linked with either DHT or a combination of EDAC and DHT to ensure that the architecture conducive for cell survival is maintained throughout the study. Following infection, scaffolds crosslinked using DHT only had a significantly higher rate of collagen degradation, than those crosslinked using the combination DHT and EDAC. This could be seen across the lower seeding density (Fig 1A–1D) and the higher seeding density (Fig 1E–1H) over 7 days (Fig 1). This result demonstrates the importance of robustly crosslinking the collagen scaffolds in order to resist degradation and preserve the porous architecture when the scaffolds are cultured with live bacteria.

**Fig 2:**
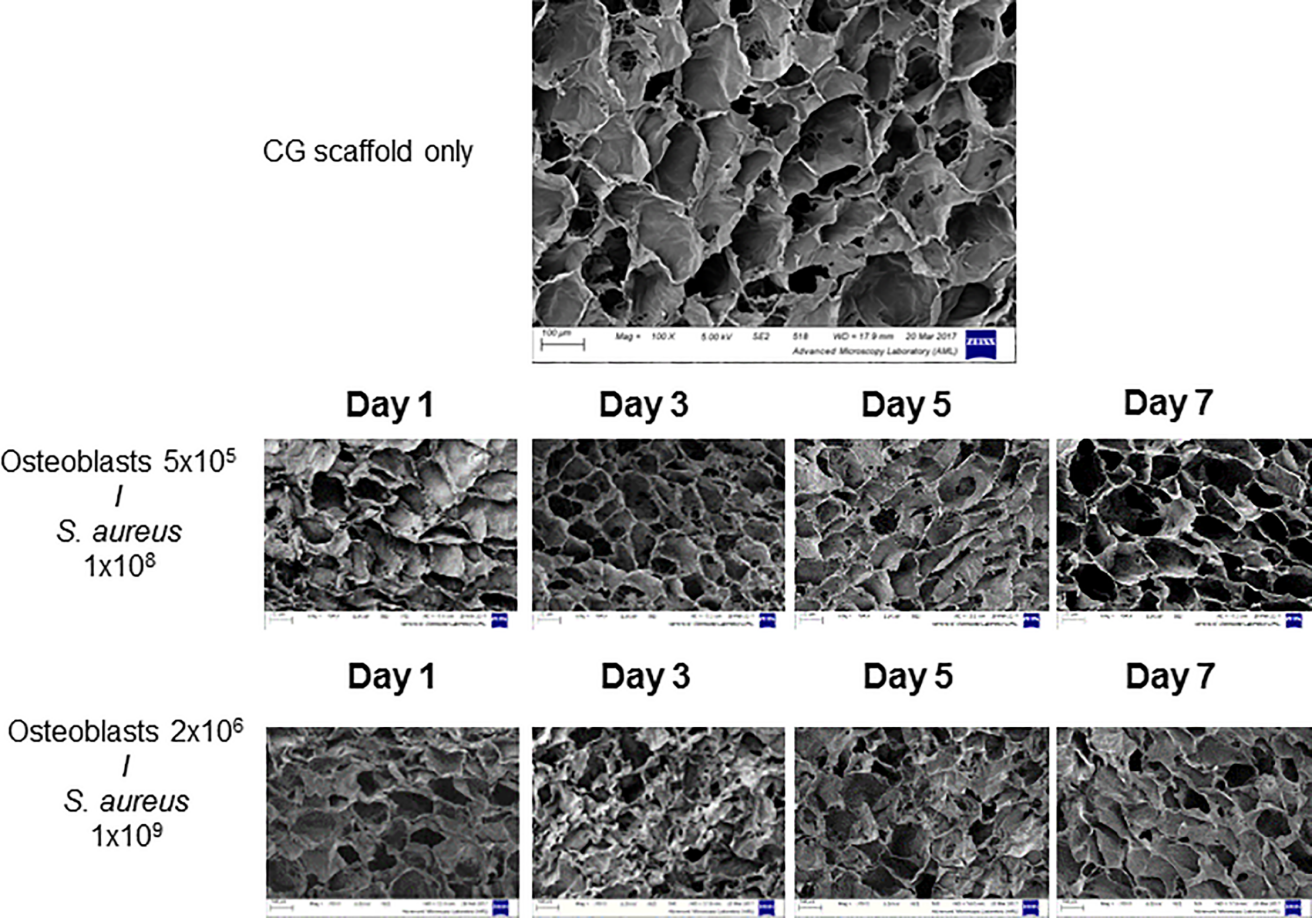
Qualitative assessment of the pore architecture using SEM of scaffolds seeded with osteoblasts and infected by *S*. *aureus* Newman. Scaffolds seeded with MC3T3-E1 5 x 10^5^ and infected with 1 x 10^8^ *S*. *aureus* Newman retained a porous structure across the 7 days of infection compared to scaffolds seeded at a higher density. Images are representative fields taken from three independent experiments that yielded similar results.

We next investigated the pore architecture in the presence of infection. Scaffolds seeded with 5x10^5^ osteoblasts and infected with 1x10^8^ *S*. *aureus* retain the original porous nature of the biomaterial. However, scaffolds infected with the higher densities of 2x10^6^ osteoblasts infected with 1x10^9^ *S*. *aureus* failed to retain the porous nature of the collagen scaffold (Fig 2). The porosity, pore interconnectivity, pore size and pore shape are all important components of the scaffold microarchitecture [27]. SEM images demonstrate that scaffolds seeded with 5 x 10^5^ osteoblasts and infected with 1 x 10^8^ *S*. *aureus* have a more open and polygonal architecture compared to scaffolds seeded with 2 x 10^6^ osteoblasts infected with 1 x 10^9^ *S*. *aureus*, which demonstrate non-uniform and non-equiaxed architecture. Thus the optimal seeding density on CG scaffolds crosslinked using DHT and EDAC is 5 x 10^5^ osteoblasts, infected with 1 x 10^8^ *S*. *aureus*. These conditions were used throughout the rest of the study.

### *Staphylococcus aureus* and osteoblasts co-infiltrate the scaffold successfully

**Fig 3:**
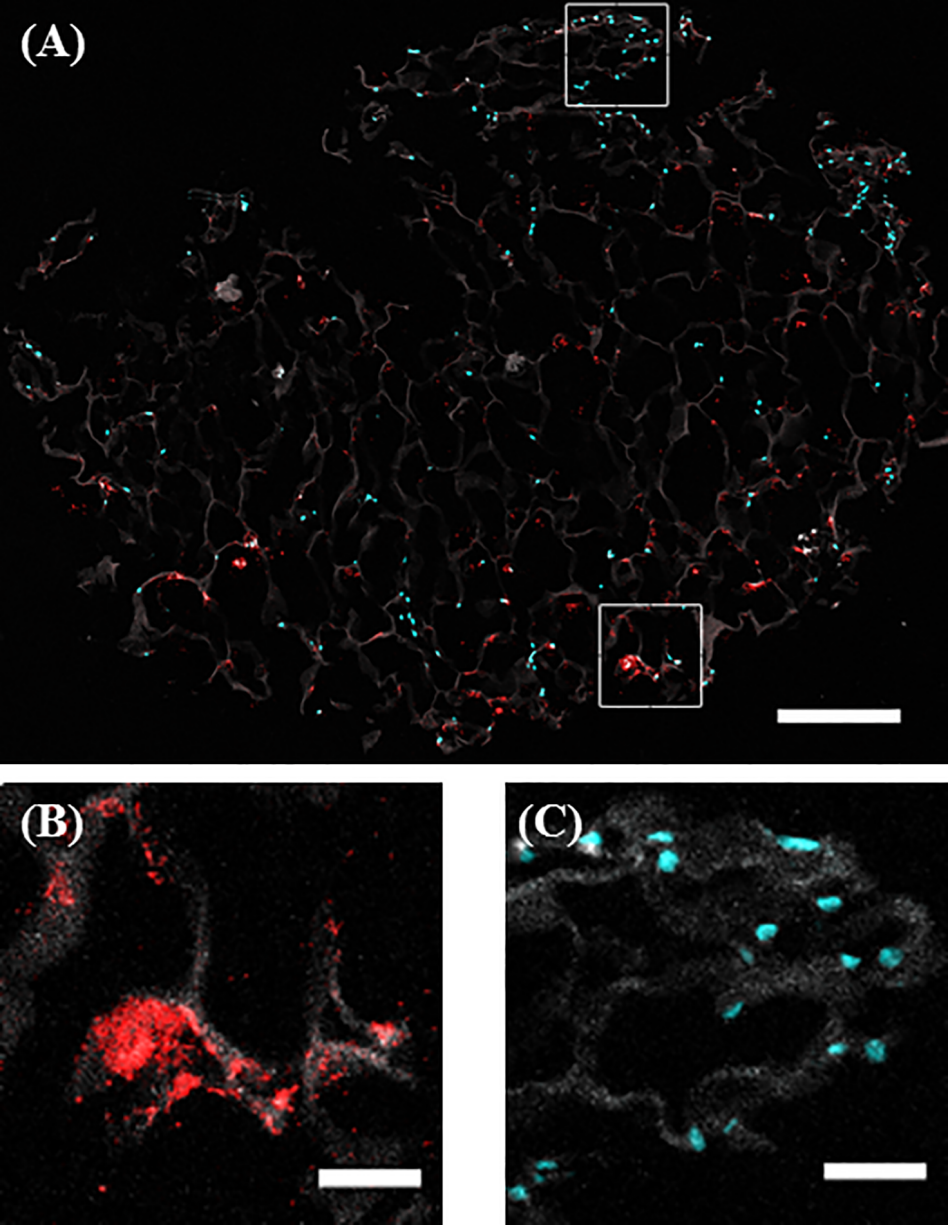
Qualitative assessment of cellular and bacterial co infiltration of the collagen scaffold. Scaffolds were seeded with 5 x 10^5^ osteoblasts (cyan) for 7 days, infected by 1 x 10^8^ *S*. *aureus* Newman (red) for 24 hr. After 24 hr, scaffolds were harvested, sectioned into 10micron sections and imaged using confocal microscopy. Overall, it can be seen that both cells and bacteria infiltrate to the centre of the scaffold (A). When zoomed in, it can be seen that both osteoblasts (B) and bacteria (C) are organised along the collagen fibres of the scaffold (10X) (B) demonstrating that *S*. *aureus* Newman interacts with both the CG scaffold and osteoblasts (100X). Scale bars are 300 μM (A), and 50 μM (B and C). Images are representative fields taken from three independent experiments that yielded similar results.

Encapsulation of the cells around the periphery of the scaffold is a common issue in 3D culture of biomaterials. Therefore to ensure that the collagen scaffolds are successfully infiltrated by both osteoblasts and *S*. *aureus*, both cells and bacteria were visualised on the scaffold using confocal microscopy. Analysis of the infected scaffolds demonstrates successful co-infiltration of both osteoblasts and *S*. *aureus* into the centre of the scaffold (Fig 3A). Both osteoblasts and *S*. *aureus* were shown to align along the collagen struts, demonstrating their attachment to the scaffold (Fig 3B and 3C, respectively). These results suggest that bacterial and osteoblast migration into the centre of the scaffold is occurring and represents the physiological conditions found at sites of infection within the bone.

### Osteoblast proliferation is significantly reduced following in the presence of *Staphylococcus aureus* when cultured in a 3D matrix

**Fig 4:**
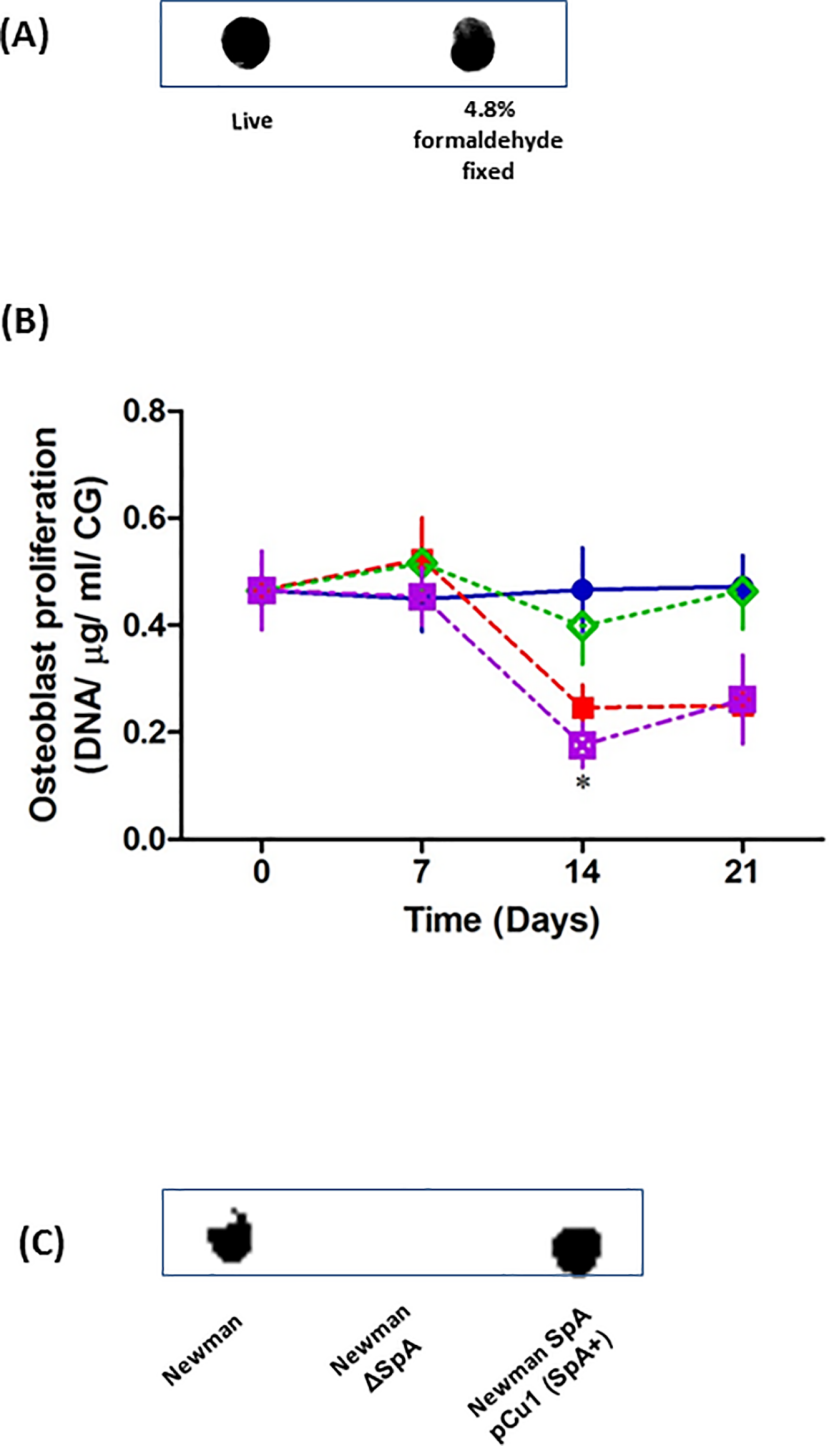
The presence / absence of SpA on the surface of *S*. *aureus* Newman WT, ΔSpA and pCu1*spa* was confirmed by dot blot (A+C, n = 3). Uninfected osteoblasts (5 x 10^5^) were seeded for 7 days on CG scaffold (Blue) and incubated with *S*. *aureus* Newman (Red), *S*. *aureus* Newman ΔspA (Green) or *S*. *aureus* Newman SpA pCu 1(SpA^+^) (Purple). Following 0, 7, 14 and 21 days post infection, remaining DNA was measured using Pico Green (B). *P < 0.05, (n = 5–17).

Having established the optimal conditions necessary to study bone infection in our 3D model, we next sought to investigate osteoblast response to the presence of *S*. *aureus*. Live *S*. *aureus* can use nutrients in tissue culture media to grow and divide. If the *S*. *aureus* use or compete with the osteoblasts for these critical growth nutrients it will prevent the growth and expansion of the cultured osteoblasts. As we are predominantly interested in the osteoblasts response to infection we fixed the *S*. *aureus* in a mild formaldehyde solution to prevent them using the essential nutrients required for osteoblast proliferation. Consistent with previous observations, mild *S*. *aureus* fixation failed to have any effect on the expression of major cell wall proteins, specifically Protein A as determined by dot blot (Fig 4A) nor did contribute or influence how the bacteria bind to osteoblasts [10].

In the absence of *S*. *aureus*, osteoblasts proliferated at a constant rate over a 21 day period. Addition of wildtype *S*. *aureus* significantly reduced osteoblast proliferation at days 14 and 21 (Fig 4B). Previously we demonstrated that *S*. *aureus* major cell wall protein, protein A (SpA), plays a key role in attachment to osteoblasts. Using a strain defective in protein A expression (ΔSpA) acted similar to the uninfected osteoblasts that failed to have any significant effect osteoblast proliferation (*P* = NS). However, complementing the ΔSpA strain with the *spa* gene (pCU1*spa*) significantly inhibited osteoblast proliferation comparable to cells in the presence of *S*. *aureus* WT (* *P* < 0.05) (Fig 4B). The presence / absence of SpA on the surface of *S*. *aureus* WT, ΔSpA and pCu1*spa* was confirmed by dot blot (Fig 4C). As the fixed bacteria are not metabolically active they do not have the ability to produce toxins or exoproteins thus suggesting that protein A plays a key role in preventing osteoblast proliferation.

### Osteoblasts are more metabolically active when infected by *Staphylococcus aureus* in a 3D matrix

**Fig 5:**
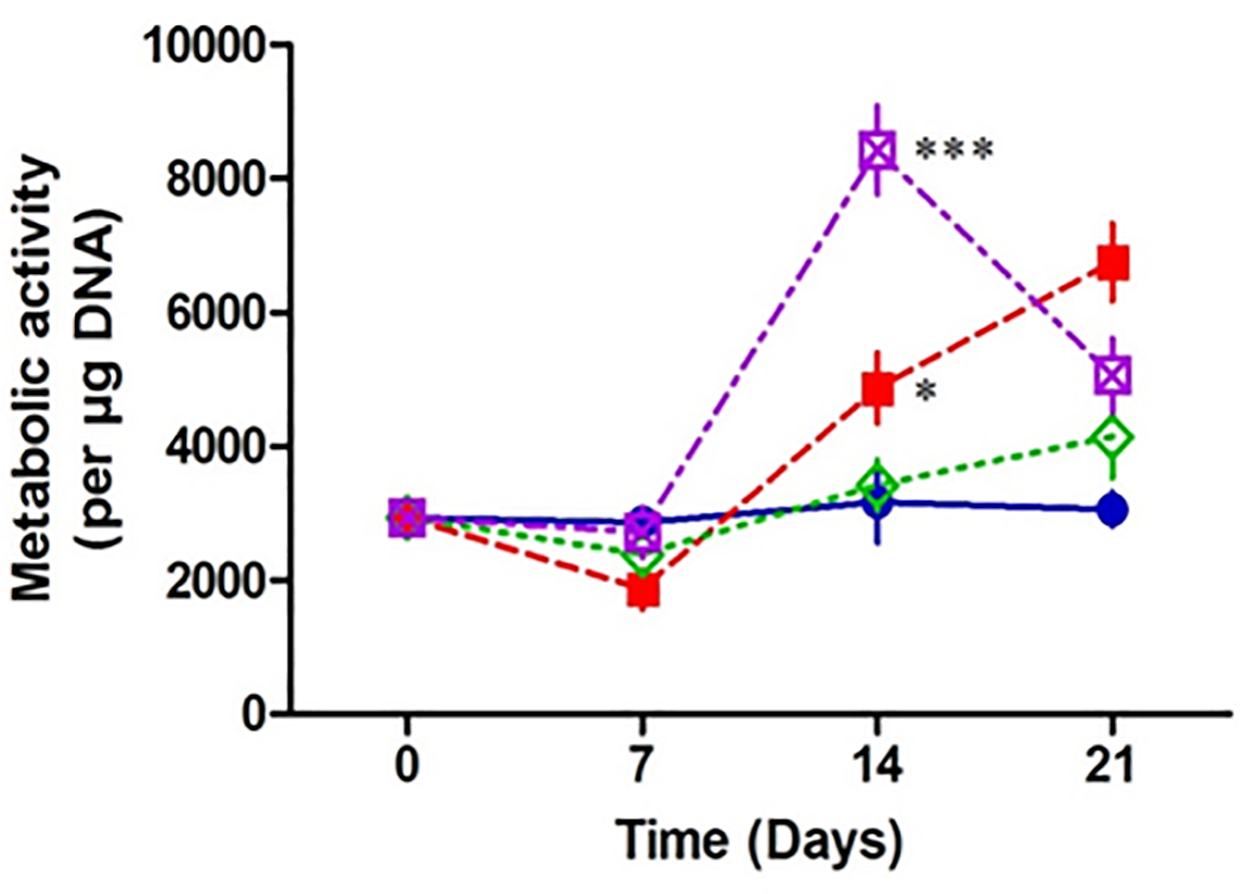
Assessment of osteoblast metabolic activity in the presence and absence of *Staphylococcus aureus*. Scaffolds were seeded with 5 x 10^5^ MC3T3-E1 for 7 days then co cultured with 1 x 10^8^ *S*. *aureus* Newman for a further 0, 7, 14 and 21 days. At each time point, Alamar blue was added to the media for 4 hr, an aliquot was removed and the fluorescent activity was measured. When normalised to the DNA content, osteoblasts co cultured with *S*. *aureus* Newman WT (red) and pCu1 (SpA+) (purple) demonstrate a significant increase in metabolic activity post 14 days compared to the control osteoblasts (blue). Osteoblasts metabolic activity is unaffected in the presence of *S*. *aureus* Newman lacking SpA (green) (B). * P < 0.05, ** P < 0.0001, (n = 3–9).

When bacteria interact with host cells, a variety of physiological responses are triggered. These responses include multiple metabolic changes in the affected host cells [9]. To investigate if the metabolic activity of osteoblasts is altered in the presence of *S*. *aureus* we measured the mitochondrial activity of the osteoblasts. Our results demonstrate that there was a significant increase in metabolic activity in osteoblasts in the presence of fixed wildtype *S*. *aureus* at days 14 and 21compared to the uninfected osteoblasts. There was no increase in metabolic activity in osteoblasts in the presence of fixed *S*. *aureus* defective in expression of SpA over the 21 day period. There was a significant increase in metabolic control in osteoblasts in the presence of fixed *S*. *aureus* ΔSpA strain complimented with the SpA gene (pCU1*spa*) at day 14 and 21, and was comparable to the increase in metabolic activity in osteoblasts in the presence of wildtype *S*. *aureus* (Fig 5). These results suggest that in the presence of *S*. *aureus*, osteoblasts have an increased metabolic activity and this is driven by SpA binding to the osteoblast.

### Osteoblasts infected by *Staphylococcus aureus* result in increased extracellular alkaline phosphatase activity

**Fig 6:**
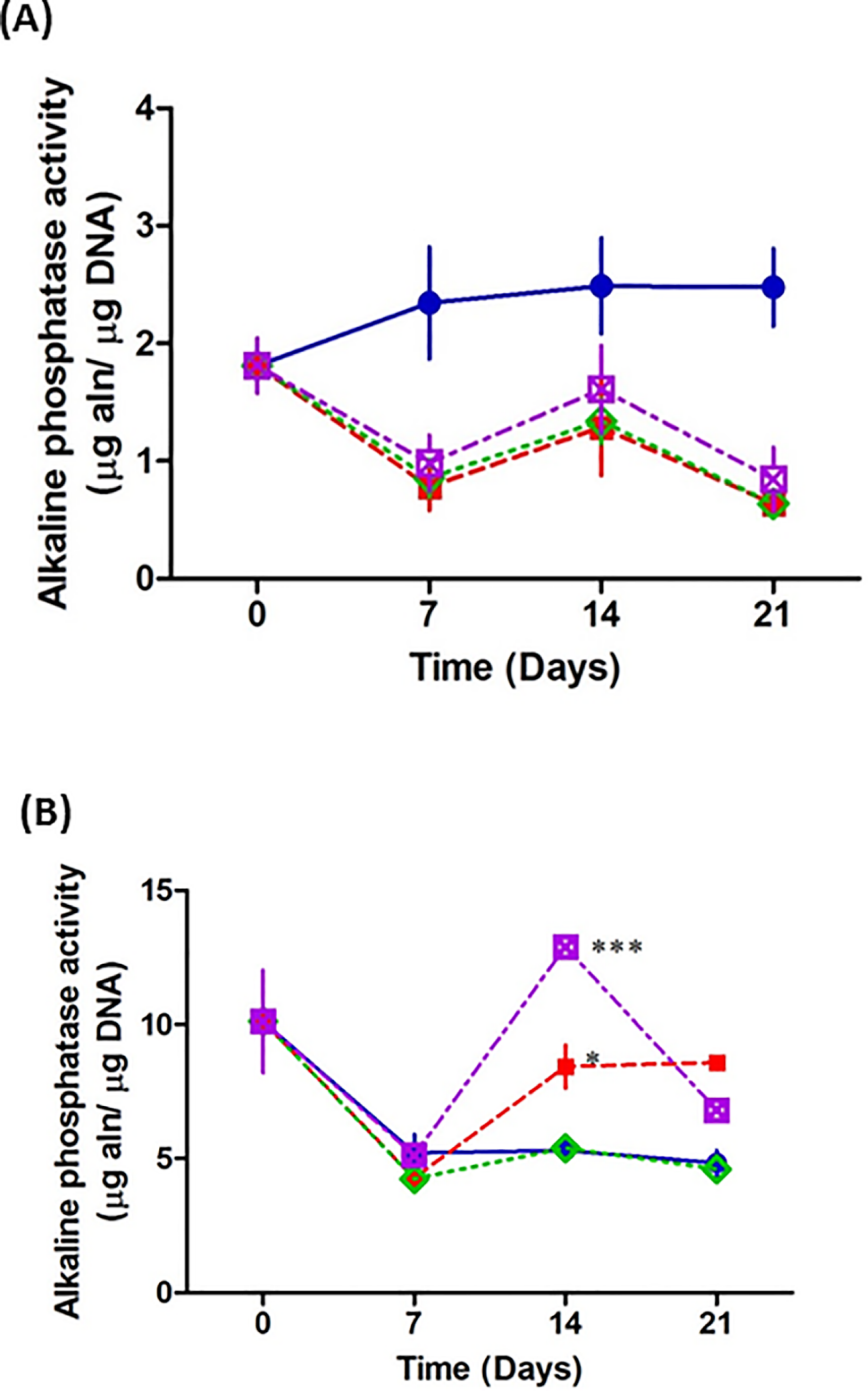
Assessment of osteogenesis in the presence and absence of *Staphylococcus aureus*. Scaffolds were seeded with 5 x 10^5^ MC3T3-E1 for 7days then co cultured with 1 x 10^8^ *S*. *aureus* Newman for a further 0, 7, 14 and 21 days. For intracellular alkaline phosphatase, at each time point, scaffolds were removed from the well, the osteoblasts on the scaffold were lysed and the lysate was incubated with PnPP. For extracellular alkaline phosphatase, an aliquot was removed from the well and incubated with PnPP. Osteoblasts cultured in the presence of all *S*. *aureus* Newman strains demonstrate a peak in intracellular alkaline phosphatase at day 14 (A). However overall, there is a reduction in intracellular alkaline phosphatase activity. Conversely, there is a significant increase in extracellular phosphatase activity in osteoblasts co cultured with *S*. *aureus* Newman WT (red) and pCu1 (SpA+) (purple) at day 14 compared to the control osteoblasts (blue). Osteoblasts extracellular alkaline phosphatase activity is unaffected in the presence of *S*. *aureus* lacking SpA (green) (B). * P < 0.05, ** P < 0.0001, (n = 5–15).

As the metabolic activity of the osteoblasts is increased following addition of *S*. *aureus*, we next investigated if this was due to osteogenic differentiation. Early bone formation is commonly measured using alkaline phosphatase, which is an enzyme found in both the membrane and cytosolic vesicles of osteoblasts [28]. Our results demonstrate that intracellular alkaline phosphatase activity is significantly decreased across all groups in the presence of fixed *S*. *aureus* over 21 days (Fig 6A). In contrast, extracellular alkaline phosphatase is significantly increased in osteoblasts in the presence of wildtype *S*. *aureus* at days 14 and 21. Osteoblasts cultured with fixed *S*. *aureus* strain defective in expression of protein A, failed to significantly affect osteoblast extracellular alkaline phosphatase activity compared to the uninfected osteoblasts. In the presence of fixed *S*. *aureus* ΔSpA strain complimented with the SpA gene (pCU1*spa*) osteoblasts had a significant increase in extracellular alkaline phosphatase activity at days 14 and 21 and was comparable to the increase in alkaline phosphatase in osteoblasts with fixed wildtype *S*. *aureus* (Fig 6B). These results suggest that in the presence of *S*. *aureus*, osteoblasts are releasing alkaline phosphatase activity and this is driven by SpA binding to the osteoblast.

### Osteoblasts produce more mineral in a 3D environment when infected by *Staphylococcus aureus*

**Fig 7:**
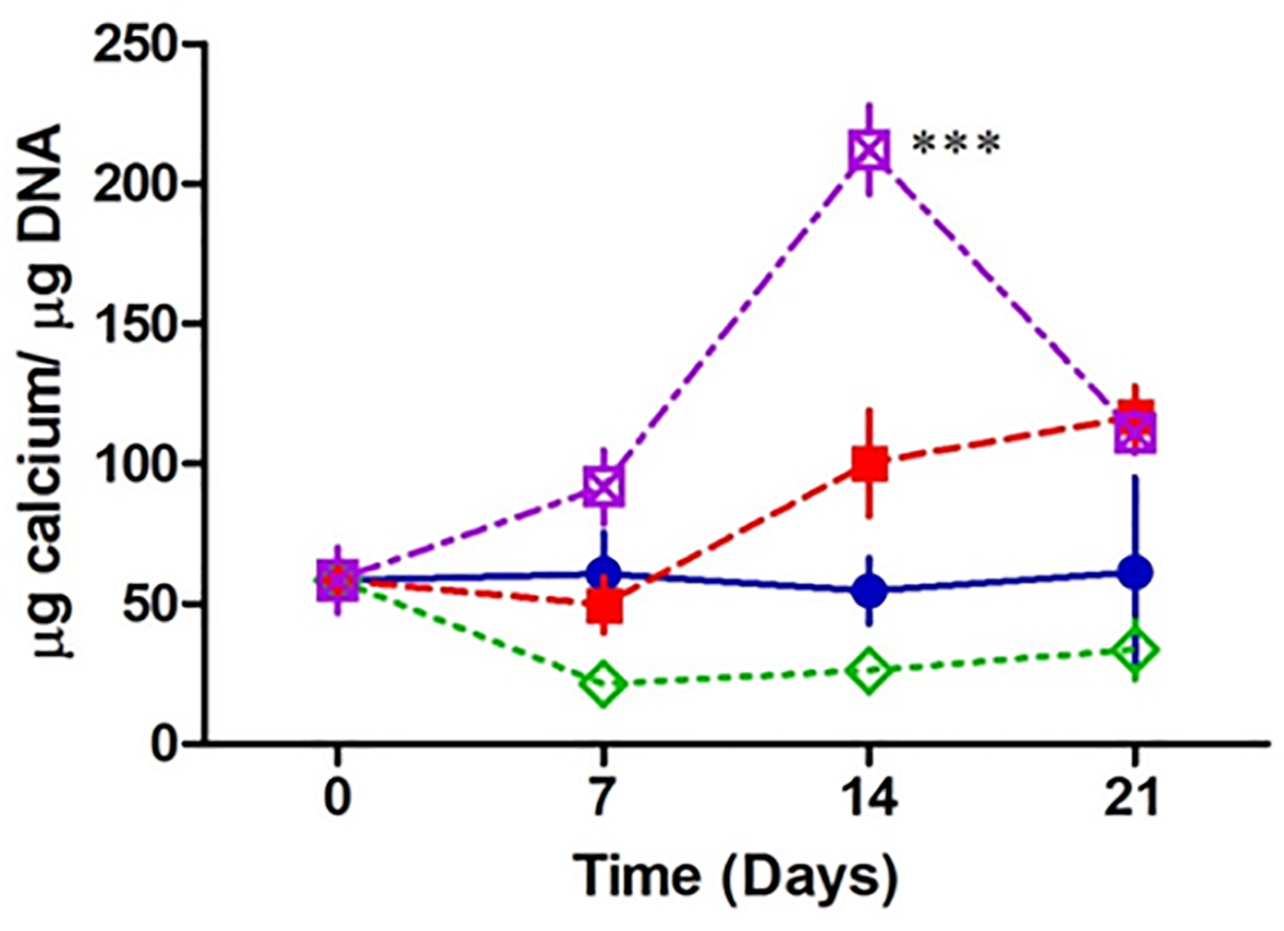
Assessment of osteoblast mineralisation in the presence and absence of *Staphylococcus aureus*. Scaffolds were seeded with 5 x 10^5^ MC3T3-E1 for 7days then co cultured with 1 x 10^8^ *S*. *aureus* Newman for a further 0, 7, 14 and 21 days. At each time point, an aliquot of supernatant was removed from the well, incubated overnight at 4°C in HCL, and the absorbance was then measured. When normalised to the DNA content, osteoblasts co cultured with *S*. *aureus* Newman WT (red) and pCu1 (SpA+) (purple) demonstrate a significant increase in metabolic activity post 14 days compared to the control osteoblasts (blue). Osteoblast mineral production is unaffected in the presence of *S*. *aureus* Newman lacking SpA (green) (A). * P < 0.05, ** P < 0.0001, (n = 3–7).

Alkaline phosphatase plays a key role in calcification. We next investigated if the increase of alkaline phosphatase leads to changes in calcification following infection. In the presence of fixed *S*. *aureus*, osteoblasts are producing significantly more calcium at day 14 and 21 compared to the uninfected osteoblasts. Osteoblasts cultured with a fixed *S*. *aureus* strain defective in expression of protein A, failed to significantly affect calcium deposition compared to the uninfected osteoblasts over the 21 day period. In the presence of fixed *S*. *aureus* ΔSpA strain complimented with the SpA gene (pCU1*spa*) osteoblasts had a significant increase in calcium deposition at days 14 and 21 and was comparable to that of wildtype *S*. *aureus* infected osteoblasts (Fig 7). These results suggest that osteoblasts are hyper mineralising the osteoblasts possibly in response to increased alkaline phosphatase release.

## Discussion

The traditional method for studying the molecular biology of bacteria bone interactions is using in vitro infections of osteoblasts or osteoclasts cultured as a two-dimensional (2D) monolayer of cells. Results presented from these 2D studies have provided critical insight into the molecular mechanisms that bacteria use to attach to and dysregulate osteoblast/osteoclast function [15]. While critical for advancing our understanding of the interactions between the cells, the nature of these experiments eludes the complex environment central to how the osteoblasts and osteoclasts develop into healthy bone giving strength and rigidity in the skeletal system [13]. To address this void, investigators have relied on a variety of animal models of osteomyelitis. While these animal models provide additional whole body responses to infection in the bone, there is a growing awareness that bacteria often behave very differently in animals as non-commensal hosts [29, 30]. This places a direct need for the development of a physiologically relevant *in vitro ex vivo* model system that better replicates the environment of the human body.

To address this we developed a three-dimensional (3D) bone infection model to examine the processes of *S*. *aureus* bone colonisation and infection. Using a well characterised collagen glycosaminoglycan biomaterial cross-linked with EDAC we co-cultured osteoblasts and *S*. *aureus* without any degradation or damage to the porous architecture of the scaffold. Both osteoblasts and *S*. *aureus* successfully migrated into the centre of the scaffold. Binding resulted in the loss of ability of the osteoblasts to proliferate over a 21 day period however most interestingly; these osteoblasts were more metabolically active than the uninfected osteoblasts. This increase in metabolic activity in the infected osteoblasts does not appear to be due to osteogenesis but rather a significant increase in calcium deposition and mineralisation on the scaffold. In order to elucidate the molecular mechanism that triggers the signals that result in these effects we used a strain of *S*. *aureus* deficient in expression of SpA, the major cell wall protein that has previously been shown to bind to osteoblast TNFR1 [10–12]. Critically none of these effects was seen in a mutant of *S*. *aureus* lacking SpA. Complementing the SpA-defective mutant with a plasmid expressing spa resulted in attachment to osteoblasts, loss of proliferation, increased metabolic rates and increased mineralisation similar to wildtype effects, suggesting SpA plays a key role in the colonisation and infection of osteoblasts in a 3D model. However most strikingly our results demonstrate a potential compensatory mechanism through which osteoblasts attempt to strengthen the bone by hyper-mineralisation as bone mass is diminished during infection.

Developing a 3D model of osteomyelitis that truly represents the physiological environment of bone requires careful attention. The use of collagen that forms the basis of a 3D scaffold is commonly used due to a number of functionally beneficial properties. Collagen scaffolds have pore microstructures that can simultaneously block cell-mediated contraction at the same time as supporting cell adhesion, proliferation, and synthesis of a functional extracellular matrix [22, 27, 31]. Reconstituted collagen has a low stiffness rate and thus fails to hold in a 3D structure when cultured with host cells including osteoblasts. To address this chemical crosslinking can be applied to enhance the chemical properties of collagen and protect it from enzymatic degradation. Our scaffolds were sterilized and dehydrothermally (DHT) crosslinked however consistent with previous observations there was significant collagen degradation following osteoblast and bacterial seeding [23, 32]. To provide further strength to the collagen, the DHT treated scaffold was further chemically crosslinked with EDAC which reduced collagen degradation resulting in improved osteoblast and bacteria migration and attachment. These conditions were used for all experiments going forward.

Significant advances have been made recently identifying the underlying pathophysiology of osteomyelitis. Current evidence suggests invading bacteria use a number of cell wall proteins to interact with host receptors on osteoblasts which lead to the generation of multiple dysregulated signals that result in bone loss and bone destruction [33, 34]. For example, *S*. *aureus* fibronectin binding proteins bind fibronectin and bridges the bacteria to the osteoblast via the fibronectin binding integrin, α5β1 [35]. Integrin clustering results in a series of cell signalling that ultimately leads to internalisation into phagocytic vesicles in the osteoblast. Once internalized, bacteria can escape the phagosome and cause osteonecrosis, causing severe weakening of the bone [36, 37]. *S*. *aureus* SpA can also bind directly to tumour necrosis factor receptor 1 (TNFR1) expressed on osteoblasts which triggers an acute inflammatory response and apoptosis which results in bone loss. At the same time there is an upregulation of RANKL, a key induction molecule involved in osteoclast activation and bone resorption which leads to bone destruction [38, 39]. Despite the significant bone loss and bone destruction, osteomyelitis patients have remarkably strong bones. In patient cases of chronic osteomyelitis, bone thickening and new bone formation reported and is mostly related to the cortical bone and periosteal lining whereby as a result of chronic infection, new bone is generated to create the involucrum which provides support to the affected bone [40]. Our results demonstrate a significant increase in metabolic rate in the infected osteoblasts. Typically a high cellular metabolic rate indicates the increased need for energy to carry out a specific cellular function often cell growth and or cell division [41]. Consistent with previous observations, we demonstrate a loss in ability of osteoblast growth or proliferation following infection, suggesting another process is being switched on, one that requires significant energy.

Mineralisation requires significant energy as it aims to deposit calcium in the bone in order to provide additional strength and rigidity in the skeletal system. Using our 3D model we demonstrate a significant increase in mineralisation in the infected osteoblasts compared to the uninfected osteoblasts. It appears that although there is significant bone loss and bone destruction during infection, osteoblasts attempt to overcome this weakness by increasing the deposition of calcium in an attempt to provide strength to the weakening bone. This finding is novel however is only seen in a 3D environment. Previously we demonstrated that in 2D cell culture conditions mineralisation was significantly reduced in *S*. *aureus* infected osteoblasts, which highlights the limitations of using 2D cell culture based models to study bone infection [10–12]. The process of increasing bone strength by increasing calcification and mineralisation also occurs in osteoporosis. Osteoporosis is the weakening of the bone due to low bone mass and microarchitectural deterioration of bone tissue with the ultimate result of weakness in the bone. Standard pharmacological intervention for osteoporosis patients is calcium supplementation in order to provide increased mineralisation, strength and rigidity in a bone where mass is significantly reduced [42, 43]. The critical difference between osteoporosis and osteomyelitis is that it appears in infected bone, osteoblasts are capable of over-compensating the bone loss by laying down more mineral in order to strengthen the bone, a role driven by SpA binding to the osteoblast.

In summary, work presented here highlights the critical role of using 3D models to improve our understanding of the pathophysiological events underlying osteomyelitis. Previously, we demonstrated that under 2D cell culture conditions, *S*. *aureus* protein A mediates attachment to osteoblasts. Upon binding there was a loss of proliferation, induction of apoptosis and inhibition of mineralization in the cultured osteoblasts in 2D. The development of collagen-based scaffolds for tissue regeneration has presented a unique focus for studying a more physiologically relevant bone infection. Using this model we confirmed that *S*. *aureus* SpA is critical for attachment to osteoblasts however in contrast with results observed in 2D, bacteria attachment resulted in hypermineralization of the osteoblasts, correlating with increased metabolic activity, when the bacteria are cultured in a 3D bone matrix. This is a critical observation that has not been demonstrated previously and aligns with the characteristic signs of osteomyelitis observed clinically. Using such 3D models will greatly improve our understanding of disease progression and thus inform our decisions for translating into *in vivo* models.

## References

[1] Lew DP, Waldvogel FA. Osteomyelitis. The New England Journal of Medicine. 1997;336(14). 10.1056/NEJM199704033361406 9077380

[2] Birt MC, Anderson DW, Bruce Toby E, Wang J. Osteomyelitis: Recent advances in pathophysiology and therapeutic strategies. Journal of Orthopaedics. 2017;14(1):45–52. doi: 10.1016/j.jor.2016.10.004 27822001 10.1016/j.jor.2016.10.004 PMC5090239

[3] Mouzopoulos G, Kanakaris NK, Kontakis G, Obakponovwe O, Townsend R, Giannoudis PV. Management of bone infections in adults: the surgeon's and microbiologist's perspectives. Injury. 2011;42:S18–S23. doi: 10.1016/S0020-1383(11)70128-0 22196905 10.1016/S0020-1383(11)70128-0

[4] Clarke B. Normal bone anatomy and physiology. Clin J Am Soc Nephrol. 2008;3 Suppl 3:S131–9. doi: 10.2215/CJN.04151206 ; PubMed Central PMCID: PMC3152283. 18988698 10.2215/CJN.04151206 PMC3152283

[5] Bouchoucha S, Drissi G, Trifa M, Saied W, Ammar C, Smida M, et al [Epidemiology of acute hematogenous osteomyelitis in children: a prospective study over a 32 months period]. Tunis Med. 2012;90(6):473–8. Epub 2012/06/14. . 22693089

[6] Penn-Barwell JG, Bennett PM, Fries CA, Kendrew JM, Midwinter MJ, Rickard RF. Severe open tibial fractures in combat trauma. Management and preliminary outcomes. 2013;95-B(1):101–5. doi: 10.1302/0301-620x.95b1.30580 23307681 10.1302/0301-620X.95B1.30580

[7] Lavery LA, Sariaya M, Ashry H, Harkless LB. Microbiology of osteomyelitis in diabetic foot infections. The Journal of Foot and Ankle Surgery. 1995;34(1):61–4. doi: 10.1016/S1067-2516(09)80103-8 7780395 10.1016/S1067-2516(09)80103-8

[8] Walter G, Kemmerer M, Kappler C, Hoffmann R. Treatment algorithms for chronic osteomyelitis. Dtsch Arztebl Int. 2012;109(14):257–64. Epub 2012/04/27. doi: 10.3238/arztebl.2012.0257 ; PubMed Central PMCID: PMCPmc3336146. 22536302 10.3238/arztebl.2012.0257 PMC3336146

[9] Brown AF, Leech JM, Rogers TR, McLoughlin RM. Staphylococcus aureus Colonization: Modulation of Host Immune Response and Impact on Human Vaccine Design. Frontiers in Immunology. 2013;4:507 doi: 10.3389/fimmu.2013.00507 PubMed PMID: PMC3884195. 24409186 10.3389/fimmu.2013.00507 PMC3884195

[10] Claro T, Widaa A, O'Seaghdha M, Miajlovic H, Foster TJ, O'Brien FJ, et al Staphylococcus aureus protein A binds to osteoblasts and triggers signals that weaken bone in osteomyelitis. PloS one. 2011;6(4):e18748 Epub 2011/04/29. doi: 10.1371/journal.pone.0018748 ; PubMed Central PMCID: PMCPmc3078117. 21525984 10.1371/journal.pone.0018748 PMC3078117

[11] Widaa A, Claro T, Foster TJ, O'Brien FJ, Kerrigan SW. Staphylococcus aureus protein A plays a critical role in mediating bone destruction and bone loss in osteomyelitis. PloS one. 2012;7(7):e40586 Epub 2012/07/14. doi: 10.1371/journal.pone.0040586 ; PubMed Central PMCID: PMCPmc3394727. 22792377 10.1371/journal.pone.0040586 PMC3394727

[12] Claro T, Widaa A, McDonnell C, Foster TJ, O'Brien FJ, Kerrigan SW. Staphylococcus aureus protein A binding to osteoblast tumour necrosis factor receptor 1 results in activation of nuclear factor kappa B and release of interleukin-6 in bone infection. Microbiology (Reading, England). 2013;159(Pt 1):147–54. Epub 2012/11/17. doi: 10.1099/mic.0.063016–0 . 23154968 10.1099/mic.0.063016-0

[13] Baker BM, Chen CS. Deconstructing the third dimension: how 3D culture microenvironments alter cellular cues. J Cell Sci. 2012;125(Pt 13):3015–24. Epub 2012/07/17. doi: 10.1242/jcs.079509 ; PubMed Central PMCID: PMCPmc3434846. 22797912 10.1242/jcs.079509 PMC3434846

[14] Fitzgerald KA, Malhotra M, Curtin CM, FJ OB, CM OD. Life in 3D is never flat: 3D models to optimise drug delivery. J Control Release. 2015;215:39–54. Epub 2015/07/30. doi: 10.1016/j.jconrel.2015.07.020 . 26220617 10.1016/j.jconrel.2015.07.020

[15] Imamura Y, Mukohara T, Shimono Y, Funakoshi Y, Chayahara N, Toyoda M, et al Comparison of 2D- and 3D-culture models as drug-testing platforms in breast cancer. Oncol Rep. 2015;33(4):1837–43. Epub 2015/01/31. doi: 10.3892/or.2015.3767 . 25634491 10.3892/or.2015.3767

[16] Popov L, Kovalski J, Grandi G, Bagnoli F, Amieva M. Three-Dimensional Human Skin Models to Understand Staphylococcus aureus Skin Colonization and Infection. Frontiers in Immunology. 2014;5(41). doi: 10.3389/fimmu.2014.00041 24567733 10.3389/fimmu.2014.00041 PMC3915142

[17] Gu L, Mooney DJ. Biomaterials and emerging anticancer therapeutics: engineering the microenvironment. Nat Rev Cancer. 2016;16(1):56–66. doi: 10.1038/nrc.2015.3 26694936 10.1038/nrc.2015.3 PMC4790726

[18] Koh W, Stratman AN, Sacharidou A, Davis GE. In vitro three dimensional collagen matrix models of endothelial lumen formation during vasculogenesis and angiogenesis. Methods Enzymol. 2008;443:83–101. Epub 2008/09/06. doi: 10.1016/S0076-6879(08)02005-3 . 18772012 10.1016/S0076-6879(08)02005-3

[19] Fitzgerald KA, Guo J, Raftery RM, Castaño IM, Curtin CM, Gooding M, et al Nanoparticle-mediated siRNA delivery assessed in a 3D co-culture model simulating prostate cancer bone metastasis. International Journal of Pharmaceutics. 2016;511(2):1058–69. doi: 10.1016/j.ijpharm.2016.07.079 27492023 10.1016/j.ijpharm.2016.07.079

[20] Ryan AJ, Brougham CM, Garciarena CD, Kerrigan SW, O’Brien FJ. Towards 3D in vitro models for the study of cardiovascular tissues and disease. Drug Discovery Today. 2016;21(9):1437–45. doi: 10.1016/j.drudis.2016.04.014 27117348 10.1016/j.drudis.2016.04.014

[21] O'Leary C, Cavanagh B, Unger RE, Kirkpatrick CJ, O'Dea S, O'Brien FJ, et al The development of a tissue-engineered tracheobronchial epithelial model using a bilayered collagen-hyaluronate scaffold. Biomaterials. 2016;85(Supplement C):111–27. https://doi.org/10.1016/j.biomaterials.2016.01.065. 26871888 10.1016/j.biomaterials.2016.01.065

[22] Haugh MG, Murphy CM, O'Brien FJ. Novel freeze-drying methods to produce a range of collagen-glycosaminoglycan scaffolds with tailored mean pore sizes. Tissue Eng Part C Methods. 2010;16(5):887–94. Epub 2009/11/12. doi: 10.1089/ten.TEC.2009.0422 . 19903089 10.1089/ten.TEC.2009.0422

[23] Haugh MG, Murphy CM, McKiernan RC, Altenbuchner C, O'Brien FJ. Crosslinking and mechanical properties significantly influence cell attachment, proliferation, and migration within collagen glycosaminoglycan scaffolds. Tissue Eng Part A. 2011;17(9–10):1201–8. Epub 2010/12/16. doi: 10.1089/ten.TEA.2010.0590 . 21155630 10.1089/ten.TEA.2010.0590

[24] Kafienah W, Sims TJ. Biochemical methods for the analysis of tissue-engineered cartilage. Methods Mol Biol. 2004;238:217–30. Epub 2004/02/19. . 14970450 10.1385/1-59259-428-x:217

[25] Ignat’eva NY, Danilov NA, Averkiev SV, Obrezkova MV, Lunin VV, Sobol’ EN. Determination of hydroxyproline in tissues and the evaluation of the collagen content of the tissues. Journal of Analytical Chemistry. 2007;62(1):51–7. doi: 10.1134/s106193480701011x

[26] Ohbayashi T, Irie A, Murakami Y, Nowak M, Potempa J, Nishimura Y, et al Degradation of fibrinogen and collagen by staphopains, cysteine proteases released from Staphylococcus aureus. Microbiology (Reading, England). 2011;157(3):786–92. doi: 10.1099/mic.0.044503–0 10.1099/mic.0.044503-0 21081759

[27] Harley BAC, Kim H-D, Zaman MH, Yannas IV, Lauffenburger DA, Gibson LJ. Microarchitecture of Three-Dimensional Scaffolds Influences Cell Migration Behavior via Junction Interactions. Biophysical Journal. 2008;95(8):4013–24. doi: 10.1529/biophysj.107.122598 18621811 10.1529/biophysj.107.122598 PMC2553126

[28] Balcerzak M, Hamade E, Zhang L, Pikula S, Azzar G, Radisson J, et al The roles of annexins and alkaline phosphatase in mineralization process. Acta biochimica Polonica. 2003;50(4):1019–38. Epub 2004/01/24. doi: 0350041019. . 14739992

[29] Patel M, Rojavin Y, Jamali AA, Wasielewski SJ, Salgado CJ. Animal Models for the Study of Osteomyelitis. Seminars in Plastic Surgery. 2009;23(2):148–54. doi: 10.1055/s-0029-1214167 PubMed PMID: PMC2884898. 20567737 10.1055/s-0029-1214167 PMC2884898

[30] Reizner W, Hunter JG, O’Malley NT, Southgate RD, Schwarz EM, Kates SL. A systematic review of animal models for Staphylococcus aureus osteomyelitis. European cells & materials. 2014;27:196–212. PubMed PMID: PMC4322679. 24668594 10.22203/ecm.v027a15 PMC4322679

[31] Keogh MB, O'Brien FJ, Daly JS. A novel collagen scaffold supports human osteogenesis—applications for bone tissue engineering. Cell Tissue Res. 2010;(340):169–77. Epub 3 March 2010. 20198386 10.1007/s00441-010-0939-y

[32] Keogh MB, O’Brien FJ, Daly JS. Substrate stiffness and contractile behaviour modulate the functional maturation of osteoblasts on a collagen–GAG scaffold. Acta Biomaterialia. 2010;6(11):4305–13. doi: 10.1016/j.actbio.2010.06.001 20570642 10.1016/j.actbio.2010.06.001

[33] Cassat James E, Hammer Neal D, Campbell JP, Benson Meredith A, Perrien Daniel S, Mrak Lara N, et al A Secreted Bacterial Protease Tailors the Staphylococcus aureus Virulence Repertoire to Modulate Bone Remodeling during Osteomyelitis. Cell Host & Microbe. 13(6):759–72. doi: 10.1016/j.chom.2013.05.003 23768499 10.1016/j.chom.2013.05.003 PMC3721972

[34] Foster TJ, Geoghegan JA, Ganesh VK, Hook M. Adhesion, invasion and evasion: the many functions of the surface proteins of Staphylococcus aureus. Nat Rev Microbiol. 2014;12(1):49–62. Epub 2013/12/18. doi: 10.1038/nrmicro3161 . 24336184 10.1038/nrmicro3161 PMC5708296

[35] Otto M. Staphylococcus colonization of the skin and antimicrobial peptides. Expert review of dermatology. 2010;5(2):183–95. doi: 10.1586/edm.10.6 PubMed PMID: PMC2867359. 20473345 10.1586/edm.10.6 PMC2867359

[36] Massey RC, Kantzanou MN, Fowler T, Day NPJ, Schofield K, Wann ER, et al Fibronectin binding protein A of *Staphylococcus aureus* has multiple, substituting, binding regions that mediate adherence to fibronectin and invasion of endothelial cells. Cellular Microbiology. 2001;3(12):839–51. 11736995 10.1046/j.1462-5822.2001.00157.x

[37] Wann ER, Gurusiddappa S, Höök M. The fibronectin-binding MSCRAMM FnbpA of *Staphylococcus aureus* is a bifunctional protein that also binds to fibrinogen. Journal of Biological Chemistry. 2000;275(18):13863 10788510 10.1074/jbc.275.18.13863

[38] Mendoza Bertelli A, Delpino MV, Lattar S, Giai C, Llana MN, Sanjuan N, et al Staphylococcus aureus protein A enhances osteoclastogenesis via TNFR1 and EGFR signaling. Biochimica et Biophysica Acta (BBA)—Molecular Basis of Disease. 2016;1862(10):1975–83. https://doi.org/10.1016/j.bbadis.2016.07.016. 27475257 10.1016/j.bbadis.2016.07.016 PMC6746341

[39] Wang Y, Liu X, Dou C, Cao Z, Liu C, Dong S, et al Staphylococcal protein A promotes osteoclastogenesis through MAPK signaling during bone infection. Journal of Cellular Physiology. 2017;232(9):2396–406. doi: 10.1002/jcp.25774 28185243 10.1002/jcp.25774 PMC5485048

[40] Levine SM, Lambiase RE, Petchprapa CN. Cortical Lesions of the Tibia: Characteristic Appearances at Conventional Radiography. RadioGraphics. 2003;23(1):157–77. doi: 10.1148/rg.231015088 12533651 10.1148/rg.231015088

[41] Metallo CM, Heiden MGV. Understanding metabolic regulation and its influence on cell physiology. Molecular cell. 2013;49(3):388–98. doi: 10.1016/j.molcel.2013.01.018 PubMed PMID: PMC3569837. 23395269 10.1016/j.molcel.2013.01.018 PMC3569837

[42] Hardcastle SA, Dieppe P, Gregson CL, Davey Smith G, Tobias JH. Osteoarthritis and bone mineral density: are strong bones bad for joints[quest]. BoneKEy Rep. 2015;4 doi: 10.1038/bonekey.2014.119 25628884 10.1038/bonekey.2014.119 PMC4303262

[43] Waarsing JH, Day JS, Verhaar JA, Ederveen AG, Weinans H. Bone loss dynamics result in trabecular alignment in aging and ovariectomized rats. J Orthop Res. 2006;24(5):926–35. Epub 2006/04/04. doi: 10.1002/jor.20063 . 16583450 10.1002/jor.20063

